# A Case Report of Juvenile-Onset Recurrent Respiratory Papillomatosis

**DOI:** 10.7759/cureus.62734

**Published:** 2024-06-19

**Authors:** Farhana Kamaruzaman, Rohaida Ibrahim, Nik Khairani Nik Mohd, Nadhirah Mohd shakri

**Affiliations:** 1 Department of Otorhinolaryngology, Hospital Sultanah Nur Zahirah, Kuala Terengganu, MYS; 2 Department of Otorhinolaryngology, Faculty of Medicine, Universiti Kebangsaan Malaysia Medical Centre, Kuala Lumpur, MYS

**Keywords:** larynx, papilloma, juvenile, recurrent respiratory papillomatosis, laryngeal papillomatosis

## Abstract

The most common benign laryngeal neoplasm in children is a papilloma. Laryngeal papillomatosis is a chronic disease and is rare in children. We report the case of a four-year-old Malay girl in whom chronic laryngeal papillomatosis, most likely acquired vertically during labor, was detected. She presented with hoarseness of voice for three years, and a flexible laryngoscopy examination revealed features of papilloma in the glottis area. The patient underwent direct laryngoscopy followed by excision of mass using the cold instrument. Surgical intervention is the primary treatment modality for laryngeal papillomatosis to maintain airway patency and voice quality.

## Introduction

Laryngeal papillomatosis, also known as recurrent respiratory papillomatosis (RRP), is a chronic disease caused by a low-risk human papillomavirus (HPV). The incidence rate in children was reported as 4.3 per 100,000 [1]. Patients under 12 years presenting with this disease show a high recurrence rate compared to adult onset [2]. Various treatment modalities have been proposed to treat this disease [2]. We report a case of juvenile-onset RRP and its management.

## Case presentation

A four-year-old girl presented with persistent hoarseness since the age of one. It was not associated with other upper aerodigestive tract symptoms such as dysphagia, odynophagia, respiratory distress, or noisy breathing. She sought treatment from general practitioners but was treated for an upper respiratory tract infection. She had a history of admissions for pneumonia, requiring non-invasive ventilation for maximum oxygen support. She was the third of four siblings, born full-term via spontaneous vaginal delivery. Further history revealed that her mother had a history of vaginal warts prior to delivery and was treated as an outpatient. There was no previous history of intubation or trauma. The examination showed no sign of respiratory distress. The neck and throat examinations were unremarkable.

**Figure 1:**
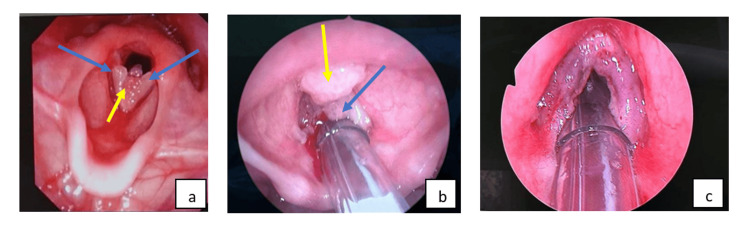
(a) Preoperative FNPLS examination revealed a wart-like growth over the bilateral vocal folds, involving the anterior commissure. Intraoperative findings showed (b) an exophytic and pedunculated mass at the bilateral vocal cord (blue arrow) extending to the anterior commissure (yellow arrow). (c) Vocal cord post excision of mass. FNPLS: flexible nasopharyngolaryngoscopy.

**Figure 2:**
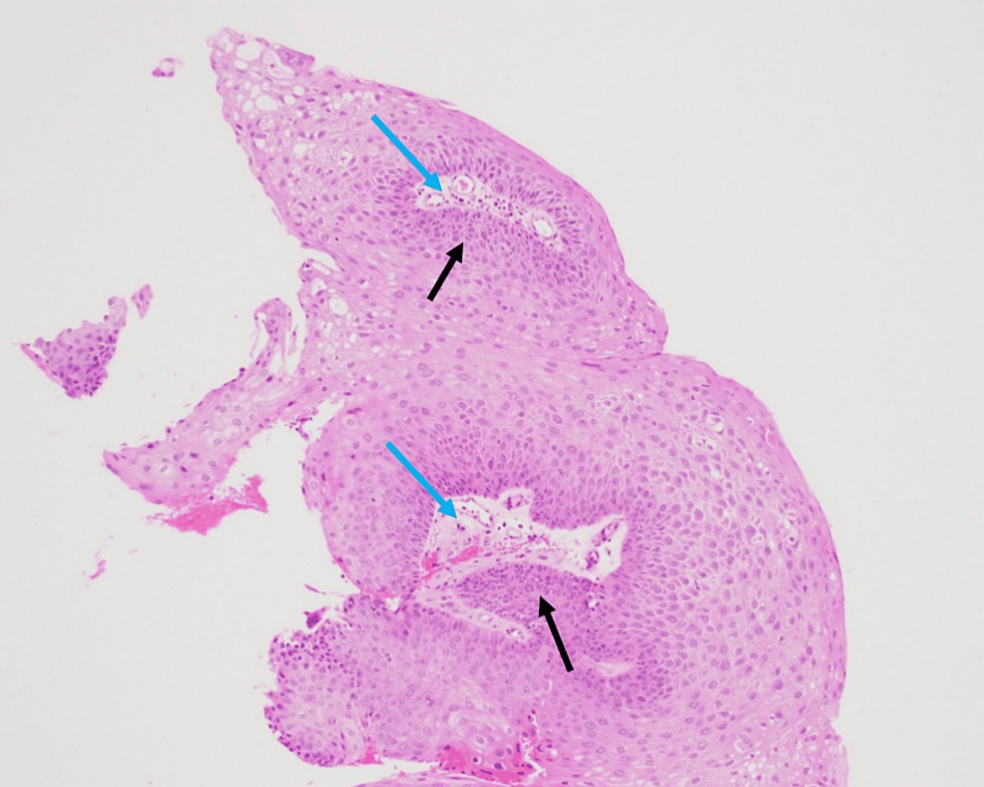
Papillary frond with a central fibrovascular core (blue arrow) covered by mature hyperplastic (black arrow), benign-looking squamous epithelium.

Flexible nasopharyngolaryngoscopy (FNPLS) examination revealed a wart-like growth over the bilateral vocal folds, involving the anterior commissure (Figure 1a). The patient subsequently underwent direct laryngoscopy and debulking of mass by using a cold instrument. Intraoperative findings showed exophytic lesions with punctate hemorrhage arising from the bilateral vocal folds involving anterior and posterior commissures and extending inferiorly to the subcordal region (Figure 1b, c). She received nebulized Ciprodex twice a day for five days, which consists of 0.5 ml Cilosan (0.3% ciprofloxacin eye drop), 0.5 ml Maxidex eye drop (0.1% dexamethasone eye drop), and 1 ml saline. Histopathological examination revealed squamous papilloma with fragments of polypoid lesion composed of papillary frond with a central fibrovascular core covered by mature hyperplastic squamous epithelium (Figure 2).

**Figure 3:**
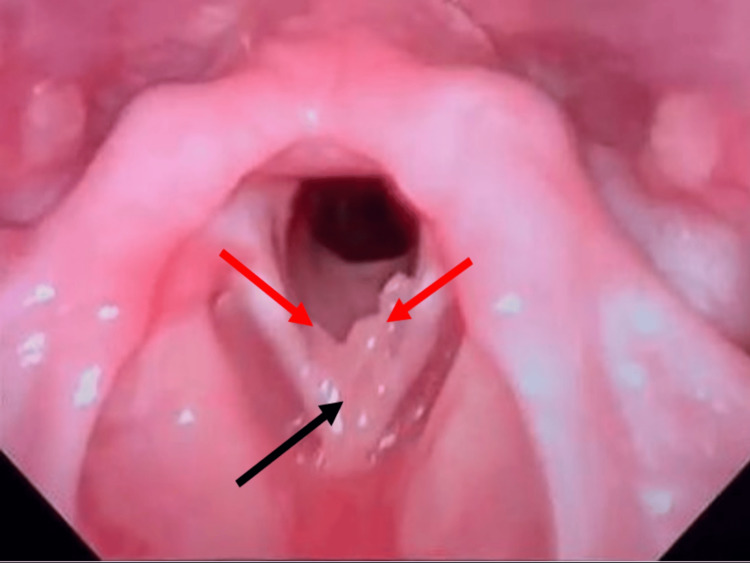
FNLPS three months postoperative; pedunculated mass at the anterior commissure (black arrow) extending to the left true cord and irregular mucosa over the right true cord (red arrow). FNPLS: flexible nasopharyngolaryngoscopy.

Follow-up three months postoperatively showed the presence of recurrence (Figure 3); however, given the improvement in her voice and no sign of respiratory distress, we continued observation and started her on syrup omeprazole. She was scheduled for a two-month follow-up to review her condition and planned to undergo another surgery for the excision of papilloma.

## Discussion

RRP is characterized by the formation of exophytic proliferative lesions of connective tissue covered by epithelium anywhere in the respiratory tract, with predilection towards the squamocolumnar junction primarily involving the larynx, especially vocal cords. There is a bimodal age distribution: juvenile-onset RRP (JORPP) occurs in children below 12 years and adult-onset RRP (AORRP) occurs after 12 years of age [3]. Human papillomavirus (HPV) is the established etiology for JORRP, with HPV types 6 and 11 being the most frequent strains. HPV types 6 and 11 are associated with condyloma acuminata [2]. Thus, children of mothers who have genital warts are at high risk of vertical transmission of HPV during vaginal delivery. The triad of JORRP is first-born, delivered vaginally, and child of a young mother [4]. In this case, despite being atypical as the patient is not the first child, the pregnancy-related condyloma is likely to be the primary risk factor for vertical HPV transmission during vaginal delivery and the acquisition of JORRP [3].

The most common clinical presentation for JORRP is progressive dysphonia, as the glottis is the most common site for RRP. Referrals to otorhinolaryngologists for FNPLS should be made for patients who exhibit persistent hoarseness of voice in primary care, as this procedure is paramount in making the clinical diagnosis, which typically reveals exophytic and pedunculated lesions with punctate hemorrhage. The largest case series in our country by Goh et al. showed 68.4% of patients had extralaryngeal involvement of RRP [3]. Thus, it is important to examine the entire airway thoroughly to exclude extralaryngeal involvement of RRP. Histologically, the papillomas are exophytic finger-like projections of stratified squamous epithelium supported by a connective tissue stroma with abnormal keratinization and basal cell hyperplasia [5].

Repetitive debulking surgery for airway control while preserving voice quality is the standard treatment for RPP. Various surgical modalities have been used in RRP cases, such as laser, microdebrider, and cold instruments [6]. Surgeons using lasers benefit from unhindered vision of the surgical field with less tissue manipulation. CO_2_ lasers have been widely used, given their cutting and cauterizing abilities. Photoangiolytic lasers have better hemostatic effects than the CO_2_ laser and better preservation of surrounding normal tissue; however, CO_2_ lasers have been reported to be less likely to cause deep tissue damage than photoangiolytic lasers [7]. Microdebrider and cold instruments are still in use for the rapid removal of lesions; however, they are unable to accurately clear in regions such as the ventricle and anterior commissure.

Choices of surgical modality are highly dependent on the surgeon’s skill and the availability of modalities in the institution. In the present case, a cold instrument was used in conjunction with a nebulized Ciprodex (ciprofloxacin and dexamethasone) and a proton pump inhibitor (PPI). Yet no previous studies have found an association between PPI and the efficacy of nebulized Ciprodex in postoperative JORRP. However, there is a study that showed using inhaled corticosteroids and PPIs immediately postoperatively following airway surgery is effective in preventing granulation tissue formation and protecting the airway from reflux, which can worsen the voice after surgery [8]. Tracheostomy is reserved for cases of acute airway obstruction and aggressive RRP. It creates a new squamocolumnar junction for HPV to breed and causes extralaryngeal spread.

The HPV genome remains in normal-appearing tissue and is the cause of recurrence. Despite surgical debulking being the standard treatment for RRP, 20% of patients require adjuvant therapy to control the disease [9]. Alanazi et al. reported that multiple adjuvant therapies have been used, including cidofovir, interferon, bevacizumab, PD-1 inhibitor, acyclovir, indole-3-carbonol, retinoid, and ribavirin [6]. Indications for initiating adjuvant therapy are patients undergoing surgery more than four times per year, rapid lesion regrowth with airway compromise, and disease spreading to the distal airway [2]. Ivancic et al. compared literature reviews on using adjuvant intralesional cidofovir. The results show 75% of JORRP patients’ complete remission after intralesional cidofovir following surgical excision [1]. However, the experience by Goh et al. using Gardasil and intralesional cidofovir was the opposite, and the patient developed recurrence after completing adjuvant therapies [3].

There is a potential that RRP will transform into dysplasia and invasive carcinoma. In RRP patients, the risk of dysplasia is about 10%, while malignant transformation is reported at 1% in JORRP and 3-7% in AORRP [10]. In contrast to HPV types 6 and 11, HPV types 16 and 18 carry a higher risk of malignant transformation. However, HPV 11 has more malignant potential compared to type 6 [6].

## Conclusions

A case of RRP is rare in children, and the primary risk factor for JORRP is pregnancy-related condylomas with vaginal delivery. JORRP is incurable; thus, repeated surgeries are required and adjuvant therapies are applied when the disease is unable to be controlled with surgery. It can be difficult to preserve voice quality in patients with JORRP following surgery since the surrounding tissue may be damaged.
